# Epilepsy & Behavior Reports thank you to our Reviewers!

**DOI:** 10.1016/j.ebr.2023.100593

**Published:** 2023-02-27

**Authors:** William O. Tatum

**Affiliations:** IV, DO FAAN, FACNS, FAES Immediate Past Editor-in-Chief, Epilepsy & Behavior Reports Professor of Neurology, Mayo Clinic College of Medicine & Health Sciences Jacksonville, Florida USA

After 7 years as Editor-in-Chief of Epilepsy & Behavior Reports (EBR) and at the start of a new year, my last word to our reviewers is THANK YOU!! We have seen unprecedented times with the COVID-19 pandemic, a time when EBR shined like a beacon of light in the dark to continue her current pathway of success. EBR has evolved from a case report journal to a high-quality peer-reviewed on-line news piece highlighting rare cases and unique patient experiences focused on the behavioral aspects of people with epilepsy. All manuscripts submitted to EBR are peer reviewed and available on PubMed. If accepted, articles are edited, abstracted for the lay population, and handled for rapid publication to readers with global access to https://www.sciencedirect.com.

EBR stimulates her readership with literature that serves to advance multinational and multicultural aspect of epilepsies on the world-wide stage. Two invited reviewers provide expert comments on a submitted manuscript. Through volunteer efforts, reviewers selflessly provide academic input to address novelty and quality of submissions devoted to the behavioral aspects of people with seizures and epilepsy. As the proud companion of Epilepsy & Behavior (Marco Mula, Editor-in-Chief) EBR, like her sister journal, publishes full-length research papers, review articles, systematic and meta-analyses, short communications, opinions and controversies, editorials and unique case reports with our fine colleagues at Elsevier. From pediatrics to adults, topics incorporate aspects involving antiseizure medications and pharmacology, electrophysiology, neurology/neurosurgery, neuroimaging, pathology, and psychiatry and psychology as the backbone of EBR. The number and quality of accepted articles continues to rise. With over 300 invited reviewers in 2022, selected specifically by their area of expertise, software helps identify authors of previously published articles all over the world. Given time limitations for volunteer reviewers, interesting titles of articles within their area of expertise are selected. This allow an expert and critical review of information that is important to ensure top-quality publication for the EBR readership. In addition, it allows initial review of new information that maybe useful to the reviewers to include as an update within one’s area of interest. Information on clinical epilepsy, surgical therapy, new antiseizure medication, semiology, intracranial EEG, neuropsychological assessment, and social issues including SUDEP have been included in accepted journal articles among many other topics. The origin of reports in EBR stem from academic epilepsy centers, communities, and rural areas in developed and developing countries from all over the world.

Our international list of reviewers and board members has been cultivated to include a strong academic placeholder for the direction of EBR during the review process. It is with humility and pride that the editors of EBR, extend our gratitude to reviewers. Those who dedicate their expertise and volunteer time to make EBR the best journal that it can be. As we begin 2023, we look forward to receiving more interesting manuscripts dealing with epilepsy and behavior. We thank our reviewers, editorial board members, Elsevier, and especially our authors for terrific and interesting contributions that provide our readers rare, unexpected, and novel findings to help understand the successes and barriers to overcome for people with epilepsy. From all of us at EBR and on behalf of Elsevier, we wish you a warm, successful, and productive new year, with health and happiness in 2023.

